# Nutritional Orthopedics and Space Nutrition as Two Sides of the Same Coin: A Scoping Review

**DOI:** 10.3390/nu13020483

**Published:** 2021-02-01

**Authors:** Matteo Briguglio

**Affiliations:** IRCCS Orthopedic Institute Galeazzi, Scientific Direction, Via Riccardo Galeazzi 4, 20161 Milano, Italy; matteo.briguglio@grupposandonato.it

**Keywords:** healthy eating, dietary supplement, musculoskeletal physiological phenomena, bones and bone tissue, sarcopenia, age-related bone losses, space travel, gravity, altered, nutritional physiological phenomena, aging prematurely

## Abstract

Since the Moon landing, nutritional research has been charged with the task of guaranteeing human health in space. In addition, nutrition applied to Orthopedics has developed in recent years, driven by the need to improve the efficiency of the treatment path by enhancing the recovery after surgery. As a result, nutritional sciences have specialized into two distinct fields of research: Nutritional Orthopedics and Space Nutrition. The former primarily deals with the nutritional requirements of old patients in hospitals, whereas the latter focuses on the varied food challenges of space travelers heading to deep space. Although they may seem disconnected, they both investigate similar nutritional issues. This scoping review shows what these two disciplines have in common, highlighting the mutual features between (1) pre-operative vs. pre-launch nutritional programs, (2) hospital-based vs. space station nutritional issues, and (3) post-discharge vs. deep space nutritional resilience. PubMed and Google Scholar were used to collect documents published from 1950 to 2020, from which 44 references were selected on Nutritional Orthopedics and 44 on Space Nutrition. Both the orthopedic patient and the astronaut were found to suffer from food insecurity, malnutrition, musculoskeletal involution, flavor/pleasure issues, fluid shifts, metabolic stresses, and isolation/confinement. Both fields of research aid the planning of demand-driven food systems and advanced nutritional approaches, like tailored diets with nutrients of interest (e.g., vitamin D and calcium). The nutritional features of orthopedic patients on Earth and of astronauts in space are undeniably related. Consequently, it is important to initiate close collaborations between orthopedic nutritionists and space experts, with the musculoskeletal-related dedications playing as common fuel.

## 1. Introduction

Over the centuries, the exploration of planet Earth required humans to venture into previously unknown territories. Food security in uncharted lands was of vital importance, and travelers had to establish agricultural or animal food systems as soon as supplies were running low [1]. This liaison between the environment and food resources is part of the nutritional paradigm that has always accompanied human survival on Earth: Environment ↔ Husbandry ↔ Food ↔ Health. Namely, the environmental conditions shape the procurement (animal or plant sources), the resulting food, and the nourishment of humans. If the *Environment* factors change, *Husbandry* and *Food* can adjust to balance human *Health*. The connection between *Food* and *Health* is of critical importance as the former has been considered not only a source of energy but also a medicine for humans, long before the first great explorations. For instance, Hellenic soldiers consumed high-iron foods to gain stamina before battles and village doctors recommended diverse dietary supplements, such as watercress or garlic, for enhancing recovery [2,3,4]. The nutritional paradigm has remained the same up to the present day: hostile surroundings need food systems that support good health, with some exceptions nevertheless (e.g., nutritional transition [5]: ↓environmental hostility, ↑engineering of husbandry, ↑food security, but ↓health). In this context, hospitals and space are normally considered unfavorable environments, with diminished food and health circumstances. Indeed, nutrition sciences have specialized in each research context to compensate for these inadequacies, thus giving birth to the sciences of Nutritional Orthopedics and Space Nutrition.

The field of nutrition applied to Orthopedics has developed in recent years, driven by the need to improve the efficiency of the treatment path by enhancing the recovery after surgery [6,7]. One of the main complexities of the field is the fact that orthopedic surgery (e.g., knee/hip replacement and spine surgery) finds its typical candidate to be an aged individual commonly suffering from osteoporosis and cardiovascular disease [8]. The hospital *Environment* further exposes the patient to stress, prolonged bed rest, and poor nutrition [9]. *Food* interventions to counteract bone and muscle loss or avert distinct nutrient deficits are, therefore, accessible (e.g., tailored diets, dietary supplements) [10]. Conversely, *Food* is almost considered an extra during pioneering space missions [11,12], because the human body acclimatizes fairly well to the short-term flights (i.e., near-Earth explorations, within 120 miles above Earth [13]). However, longer voyages (i.e., interplanetary or orbital explorations, with trips lasting from weeks to years) heading to deep space expose humans to an insecure food system, osteosarcopenia, and malnutrition, all leading to difficulties in the rehabilitation after landing [14]. At zero gravity, the *Environment* factor directly affects *Food* and *Health*, the liquids break into small drops, solid foods tend to form dust (e.g., original food for space flights included bite-sized cubes and dense liquids stuffed in tubes), the body floats, and gastro-intestinal function is close to being dysfunctional [15]. Advanced food systems are to be planned for space, thus avoiding current feedback loops on Earth that lead to pollution, destruction of habitats, natural resources, and loss of biodiversity [16]). At the time when we aspire to send people to distant planets, space food research has never been more important.

Some of the above-mentioned information already anticipates some similarities between the two fields of research, orthopedic patient vs. astronaut, hospital vs. space. However, it is indistinct in the existing literature about what kind of situations orthopedic patients and astronauts share, suffer from, and what similar hazards undermine their physical and mental health. To describe these issues, a scoping review was conducted to examine the nature of the interests and evidence common to Nutritional Orthopedics and Space Nutrition, both grounded on the millennia-old nutritional paradigm mentioned above. Specifically, the question to be answered is “What is known about the commonalities between Nutritional Orthopedics and Space Nutrition?”. The query was addressed following the subdivision of each research area into parallel phases: (1) pre-operative vs. pre-launch nutrition, (2) hospital-based vs. space station nutritional issues, and (3) post-discharge vs. deep space nutritional resilience. The existing knowledge guiding the path of care of orthopedic patients before/after surgery was mapped in parallel to specular phases of space research, being the pre-launch programs and nutritional support for short/long-lasting stay in space. The description of environmental hazards and illnesses was also part of the scoping review.

## 2. Methods

**Figure A1:**
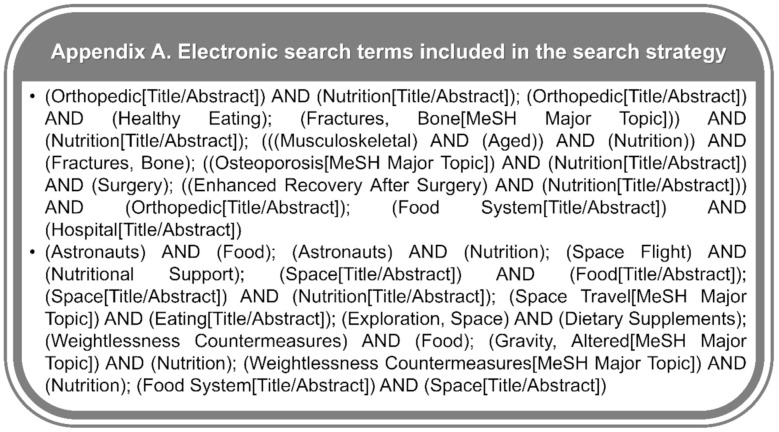
The final online search strategy for PubMed.

The review was conducted using the PRISMA−ScR (Preferred Reporting Items for Systematic Reviews and Meta-Analyses−Extension for Scoping Reviews; Supplementary Table S1; equator-network.org/reporting-guidelines/prisma-scr/), with no review protocol being currently available. Criteria for document inclusion comprised: the focus on orthopedic surgery or space travel, the discussion of nutritional issues, the development of the topic according to health-related frameworks for human survival, year of publication between 1950 and 2020, and text in English. Online sources covered PubMed and Google Scholar through a research strategy reported in Appendix A (Figure A1**)** (date of the most recent search: 22 September 2020). Identified terms were changed appropriately from PubMed to Google Scholar databases. Final documents were exported and duplicates were removed. The records were integrated by searching authoritative online sources (e.g., ntrs.nasa.gov). The process for selecting the evidence sources was conducted by a single experienced librarian who screened, charted, and abstracted independently. To ensure relevance to nutritional sciences for orthopedic (old) patients or astronauts, the primary selection focused on the words in titles/abstracts and then full texts, with the consideration of any type of document, including but not limited to animal/human studies, non-systematic and systematic reviews, meta-analyses, expert opinions, brief research/technical reports, commentaries, and press releases. This scoping review focused on the broad commonalities and, therefore, no specific type of article was excluded from the selection process. Eligible records were reported according to the year of study, study design, population, findings, and data were examined according to the relevant information useful to answer the study query. Specifically, the following data were extracted (orthopedic surgery vs. space travel): the existence and nature of nutritional support programs, the existing/desirable food systems, the existence and nature of environmental hazards, the need for particular nutrients that guarantee nutritional resilience, and the existence and nature of future research directions. No critical appraisal was conducted. To answer the review question, the range of evidence was grouped and narratively synthesized according to the following sections: nutritional needs and interventions before elective orthopedic surgery (home-based) vs. before space launch (Earth-based); the food procurement systems in hospitals vs. in space; research directions in the field of food science and nutrition in hospitals vs. in space; the hazards to human survival in a hospital vs. in space; the nutritional issues after hospitals vs. space deconditioning. Significant information on Nutritional Orthopedics and Space Nutrition is visually represented in Appendix C (Figure A3) and Appendix D (Figure A4), respectively.

## 3. Results

**Figure A2:**
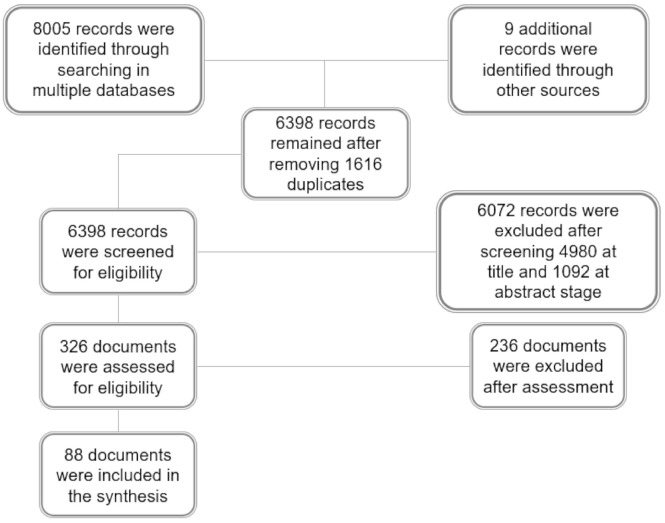
Selection process of sources of evidence on Nutritional Orthopedics and Space Nutrition.

**Figure A3:**
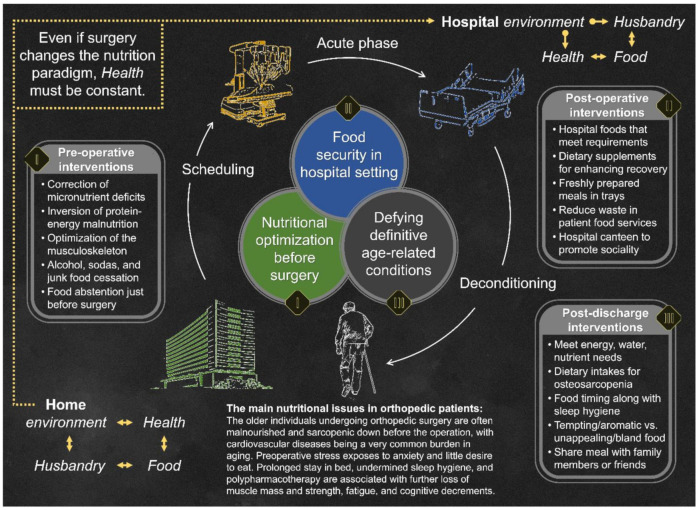
Key aspects of human nutrition in orthopedic patients. The perioperative nutritional support program for patients undergoing major orthopedic surgery comprises three phases. (I) First, the optimization of the nutritional status should indicate recommendations on healthy eating, tailored diets, and dietary supplements for nutrient deficits through a structured behavioral program. (II) Second, early oral feeding should be promoted to avoid fasting and to boost recovery. Hospital diets may be integrated according to the patient’s requirements, with services of food and beverages to patients following a model redesign to facilitate engagement and improve satisfaction. Case-specific prevention of food waste should be applied by promoting interaction in the order process and meal sharing. (III) Third, the variety of nutritional issues after discharge include not only high-risk pre-existing illnesses like frailty but also newly occurring conditions from hospital-associated deconditioning, like sarcopenia from immobility. Since the hospital could easily be considered a hostile *Environment* that affects the *Health* of patients and limits the *Husbandry* factor, it is necessary to reach a balance by acting on the central components of the nutritional paradigm, where *Food* really plays a central role.

**Figure A4:**
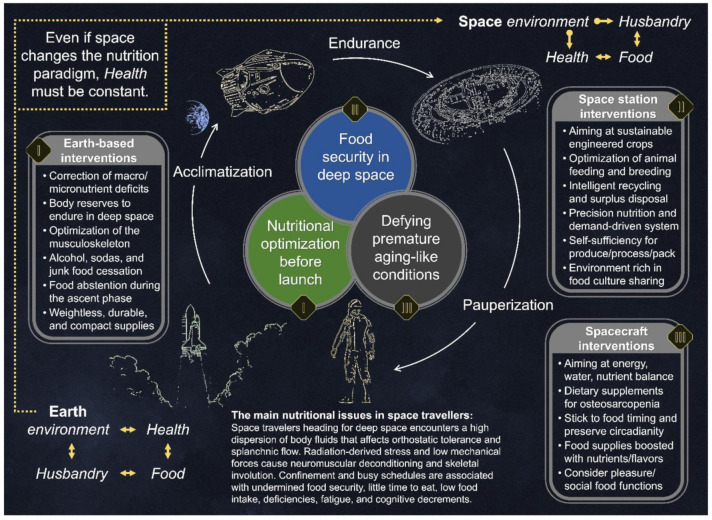
Key aspects of human nutrition during space travel. The nutritional program aiming at supporting all the individual’s activities related to space travel should cover three main aspects. (I) First, the individual’s nutritional status should be optimized before launch. Environmental conditions, especially terrestrial gravity, are able to maximize psycho- and physiological interventions. (II) Second, an autonomous food system should be settled on space or planetary stations. Advanced plantations following the principles of eco-sustainability and precision nutrition might cohabit with farms of animals with a high feed conversion efficiency, such as insects. (III) Third, Space Nutrition research should focus on issues of venturing into deep space. During the prolonged absence of the gravitational system, the human body is subjected to various senile-like conditions, which are not necessarily caused by dietary factors, but that can be defied or at least delayed by integrated approaches comprising tailored nutrition. The nutritional paradigm initiated on Earth, where human beings have found a way to make the most of it, will undergo a drastic revolution when the *Environment* factor is the deep space. Since human *Health* is assumed to be constant, environmental deviations must be balanced with novel *Husbandry* systems and *Foods*.

After the online search, a total of 44 documents (42 peer-reviewed articles + 2 book chapters [17,18]) on Nutritional Orthopedics and 44 documents (37 peer-reviewed articles + 6 documents ([19,20,21,22,23,24]) from the National Aeronautics and Space Administration + 1 document [25] from the Federal Aviation Administration (FAA)) on Space Nutrition were selected (see Appendix B (Figure A2) for the flow diagram). The scoped nutritional commonalities between the two fields of research, with relevance to the specific areas of nutritional interest mentioned above, were narratively described in the next passages (see Appendix E (Table A1)). Further details of all documents are reported in Appendix F (Table A2).

### 3.1. Pre-Operative (Home-Based) vs. Pre-Launch (Earth-Based) Nutritional Issues

#### 3.1.1. Nutritional Program Prior to Elective Orthopedic Surgery

In the mid-1990s, a state of malnutrition was already recognized as a risk for negative outcomes in surgical patients [26]. Malnutrition in Orthopedics may be detected through biochemical, anthropometric, functional, or dietetic assessments [10]. Nonetheless, it is sometimes preferred to adopt a population medicine approach in which all patients undergo nutritional optimization, regardless of their nutritional status. In fact, some age-related comorbidities and medications could inevitably worsen micronutrient reservoirs during the period up to surgery, even in the presence of a good nutritional status at the evaluation prior to admission [27]. Some patients may necessitate different routes of nutrition administration, depending on the ability to chew or on the gastrointestinal function. Enteral, parenteral, or modified texture foods are considered valuable options to attain the best possible condition before surgery.

Contrariwise, the significance of food abstention immediately before surgery is one of the most debated tenets in the field of pre-operative nutritional programs. On the one hand, there appears to be no general association with vomiting or aspiration but, on the other hand, there is a lack of available data on individual surgical procedures [28,29]. Beyond the habit of draw indications from parallel extrapolations, replacing the absolute fasting from midnight with a calorie-loaded supplement up to a few hours before surgery may be a safe element for perioperative care as far as Orthopedics is concerned [30,31].

Some deficiency syndromes, such as iron deficiency and hypovitaminosis D, are a very common burden in aged orthopedic patients and engineered formulas that facilitate intestinal absorption are therefore used (e.g., oil-in-water emulsion with propylene glycol for vitamin D [8] and sucrosomial matrix for iron [27]). Proper perioperative interventions should comprise diet and supplements tailored according to age, sex, and disease conditions, integrated with physical training, psychological support, smoking cessation, and alcohol reduction [32,33,34]. In orthopedic patients, the body composition should not only favor lean mass formation, which is vital for muscle and bone components, but also guarantee adequate fat reserves that harbor a source of energy, protect bones from traumas, and store lipophilic vitamins [35,36]. Some micronutrients, such as vitamin A and E, play major roles in the proper functioning of the immune system [37], and deficiencies should be corrected especially in aged individuals who are ordinarily affected by immuno-senescence and wound healing defects [38].

#### 3.1.2. Nutritional Conditioning Prior to Launch and “Packed Launch”

In the mid-1900s, it was already known that the astronauts must be fit to travel [39]. After all, “Finisque ab origine pendet” (Astronomica of Mark Manilius—first century C.E.). Therefore, regardless of the length of the expedition, space travelers should be subjected to nutritional optimization before departure. The goal is to achieve good body reservoirs, to avoid deficiencies of vitamins or minerals, and to obtain nutrition-derived immunocompetence from tailored supplements, such as omega-3 fatty acids [40]. Tailored dietary prescriptions and intake monitoring start months before launch, with astronauts being able to select their own space menu [19]. This personalization is known to guarantee the maximization of both physical and mental status [41].

Space travelers leave behind earthly cravings that are either nutritionally poor or counterproductive, such as junk foods, sodas, or alcoholic beverages. For example, carbon dioxide bubbles do not float in microgravity and remain in the beverage even after drinking, forming foam in the intestine, whereas ethyl alcohol is known to increase susceptibility to disorientation, to reduce reaction time and tolerance to g-forces, to impair physical performance and mental judgment [25]. When the launch day arrives, space travelers should refrain from eating food immediately prior to launch as the thrusting into microgravity is known to cause stomach awareness, nausea, and vomiting [20].

Space food should be mostly freeze-dried in individual packages to reduce water activity and help consumption. Furthermore, each package must include an adhesion system, like Velcro, to prevent it from floating. Space food should also be compact in order to take up little space (cargo capacity in space flights is limited) and to prevent dispersion due to microgravity once the package is opened [42]. The surface tension that forms with rehydration is useful to prevent the crumbs from flaking. Overall, the ultimate success of a space mission relies on food safety and stability, which collectively refer to the long-term preservation of microbiological, sensory (color, taste, flavor, and texture), and nutritional characteristics [42]. Historically, inadequate provisions are known to lead to ill-fated expeditions [43]. Since it is not feasible to overload the spaceship with food supplies and it is not convenient to keep resupplying the crew beyond near-orbit stations [44], astronauts will have to start growing their own food.

Summary I. A total of 13 references ([8,10,19,20,25,27,29,33,34,38,39,40,42]) were used to scope the following nutritional commonalities between pre-operative (home-based) vs. pre-launch (Earth-based) issues. The relevance of having good nutritional status; the importance to acquire nutrition-derived immunocompetence; the significance of food abstention before stressful situations; the goal of giving up/reducing bad habits; the need to opt for tailored nutrition and advanced dietary preparations.

### 3.2. Post-Operative (Hospital-Based) vs. Space Station (Planetary) Nutritional Issues

#### 3.2.1. Hospital Food Systems: Every Day Your Food Tray

Hospitals can adopt two main types of foodservice models according to clinical and organizational measures [45]:

- “Cook and Chill Systems”: outsourced catering systems that prepare, pack, and ship single-serving meals to the hospital, which is therefore equipped with a minimal-functionality kitchen.
- “Cook and Serve Systems”: in-house fully operational kitchen that processes and serves the meals directly to the wards.

A mixed model, with both precooked and fresh food, involves the meal transport to the ward and its cooking by using microwave ovens, allowing patients to order meals up to two hours prior to meal-times [46]. In the case of in-house kitchens, the staff provides food to different consumers, such as patients and employees, and, therefore, high amounts of food are produced simultaneously by sharing resources and equipment. However, this model is preferable because it allows better management, for example, of last-minute orders. At the same time, the kitchen should be well organized with separated compartments for storage in different environments and with spacious areas to avoid cross-contamination. Meals brought from the kitchen must be delivered on separate trays in packaging suitable for avoiding the partitioning of chemical compounds into food [47]. The patient should be able to wash his hands before meal consumption and food has to be at the right temperature. The control of the process should be as far upstream in the system as possible through continuous monitoring techniques. The modern food risk analysis and control procedures were pioneered in the 1960s for the production of safe foods for the United States space program [48]. Additionally, it should be considered that a meal’s value also depends on the quality of raw materials concerning sensory characteristics, purity, contamination, radioactivity, adulteration, and loss on drying [49]. The engagement between the patient and the meal order staff should be improved through a bedside spoken meal ordering system [50], being the productivity of the entire foodservice model primarily relied at the point of food consumption. In fact, plate waste is the major determinant of foodservice model losses [51].

#### 3.2.2. Food Research Laboratory in Hospital

Special diets for food intolerances, allergies, nutrition-related diseases, and dietary/religious dogmas should be handled by the clinical nutrition unit, working in parallel with the research laboratory, with the aim of evaluating the effectiveness of dietary interventions. Clinical- and patient-oriented outcomes should complement the “what, when, and how”, the decision-making algorithms, and the cost analysis [10,52]. Several research objectives are worth pursuing. The obstacles to early feeding such as the motion sickness exacerbating nausea/vomiting, should be investigated, considering that a diverse combination of medications might be associated with different symptoms [7]. Patients suffering from osteosarcopenia may benefit from calcium and protein supplements [53], preparations of vitamin D and iron can optimize cardiac function and iron status [27,54], and the Mediterranean diet may slow down the age-related joint degeneration and subchondral bone deterioration [55,56]. Food preservation methods are crucial for food safety and quality. Heat remains the most widely used inactivation technique, but pressure or magnetic/electric field applications are also promising [57,58]. These operations should be adjusted according to performance standards (e.g., microbial inactivation, retention of vitamins). Older orthopedic patients should be presented with appetizing meals that stimulate the age-related decay of smell, taste, and hedonic appreciations [59,60]. Ready-to-use flavor enhancers may be used to improve intakes [61], possibly reducing plate waste. Food surplus may also be associated with other causes nevertheless, such as poor meal quality and early satiety [62]. Overall, the collaboration among the foodservice, the clinical nutrition unit, and the research laboratory is certainly fundamental in order to guarantee a real benefit to hospitalized patients [63].

#### 3.2.3. Space Food Systems: Where the Light Source Is, the “Onion Spur” Grows

Any habitable space food system should be adapted to environmental conditions. Currently, two options are on the table [64].

- “Transit Food Systems”: on-orbit food systems in microgravity are based on groundbreaking operations that include prepackaged supplies and low-volume storage.
- “Planetary Surface Food Systems”: partial gravity systems, such as those that are built on other planets, sustain Earth-like processes and include plant crops and animal farms.

Low gravity shapes the turgor of plant structures, altering the physiological responses. However, long-term persistence in space can generate new cultivars and ecotypes that are more resistant, as well as new subspecies and varieties with morphological advantages [65]. Common space-related constraints for aeroponic/hydroponic crops are the proper running of irrigation systems and nutrient pumping, air regeneration, water recycling, and the cultivation area [66,67]. All aspects should be integrated by innovative systems (e.g., the Passive Orbital Nutrient Delivery System or PONDS [21]). Light-emitting diodes replace the sunlight [68], as direct solar flares from the Orion Arm, along with other galactic radiations, are known to cause severe consequences, like DNA breaks, chromosomal aberrations, genome instability, and carcinogenesis [69]. Any animal husbandries are influenced by component choices analogous to vegetable crops. Naturally, the quality of the animal product depends on the nutritional content of the feed [70]. Regrettably, the nutritional loss from this transformation remains one of the most important factors on Earth that worsens system efficiency [71]. In the 1980s, insects were suggested as valuable food for space cooking [22], in alternative to large animals (to be excluded due to their excessive consumption of resources). Insect farms take up little space, provide a steady stream of high-quality proteins, and allow efficient conversion of waste into food. For instance, the black soldier fly is known to considerably reduce the environmental impact of food production [72]. Microgravity could create some problems to flying insects, making the larvae the primary choice [73].

#### 3.2.4. Food Research Laboratory in Space

Planetary stations are likely to benefit from the presence of food research laboratories. The broad spectrum of food systems will be investigated through advanced technologies for compact food stowage, analyses for quality assurance, heat sealing and vacuum packaging for long-term shelf life, and menu design for a variety of food choices and nutritionally complete plans [23,64]. Anticipated recycling should balance surplus disposal, without leading to the abovementioned environmental degradation observed on Earth. Current examples of research centers to solve the challenges of food in space comprise the Kennedy Space Center (NASA) aiming for the exploration of Mars [74] or the Space FoodSphere (Japan Aerospace Exploration Agency or JAXA) aiming for the exploration of the Moon [75]. Cutting-edge research aims to find new plant species that do not encounter microgravity- or radiation-derived inefficiencies like lettuce [76] and duckweed [77], or biofortification techniques through cellular agriculture methods to enrich space food with molecules of interest [78]. The quantity of certain nutrients, such as vitamin K and C, may be low before packing or are known to degrade to inadequate levels over time [79]. Consequently, nutritional stability should be required to enable long-term missions. The biological role of diverse nutritional, nutraceutical, and aromatic iotas is worthy of being studied, considering that beneficial components can be incorporated directly into new chemotypes [2,80,81]. For instance, space travelers will need extra calcium and vitamin D for bone weakening, the proper amount of proteins to counteract sarcopenia, low iron to avoid overload, and low-sulfur protein intake for acid-base balancing [54,82,83,84,85]. Extra flavor molecules to increase food enjoyment may be necessary because astronauts may experience a dulling of the olfaction that is known to reduce food intake [86]. This is probably due to nasal congestion (the bodily fluids move to the upper body [20,87]) or to the fact that aromas diffuse differently in the space environment. Novel packaging techniques should fulfill the needs of food preservation, sustainable use of space resources, and strategical area assignments (e.g., the area for meal socialization during homesickness) [67,88,89]. Altogether, the integrated activities among these facilities certainly lay the foundations for a successful mission into deep space.

Summary II. A total of 33 references ([7,10,20,21,22,23,27,45,46,50,51,52,53,54,55,56,59,60,61,62,63,64,66,67,68,73,76,77,79,82,83,84,85]) were used to scope the following nutritional commonalities between post-operative (hospital-based) vs. space station (planetary) issues. The use of single-serving prepackaged meals; the need to establish demand-driven systems that crosstalk and reduce food waste; the importance of guaranteeing food safety and human engagement throughout the process; the need to support the musculoskeletal health; the interest in some nutrients (i.e., proteins, calcium, iron, vitamin D); the need for boosting food flavors to increase appetite, palatability, hedonic appreciation, and food intake.

### 3.3. Hospital vs. Deep Space Hazards and Illnesses

#### 3.3.1. Bedrest, Acute Stress, and Isolation

Aging is intrinsically associated with dietary impoverishment, musculoskeletal involution, restlessness, and progressive reduction of the ability to cope with physical and psychological stress factors [90,91]. This reduced resilience tends to worsen during prolonged hospitalization, resulting in the so-called hospital-associated deconditioning [92,93]. The first studies on the effects of prolonged immobility were advanced by the United States space agency in the 1970s [94,95]. Currently, enhanced recovery protocols tend to be fast-tracks, thus emphasizing the short hospital stay compared to classic care pathways [10]. Many hospital-associated hazards are known to cause this hospital-associated deconditioning, including:

- Bedrest. When the body is lying down, upright valves of the vascular system do not minimize the gravity-associated fluid shifts. The plasma volume decreases and affects circulation [96], increasing the risk of syncope-related falls [97]. Prolonged immobility accelerates bone and muscle loss, exposing to osteosarcopenia and increased risk of fall-related fractures [97,98]. Immobility is also associated with gastrointestinal affections, such as gastroesophageal reflux, low appetite, slow peristaltic rate, and constipation [17].
- Acute stress. Surgical incision elicits both local (e.g., tissue injury, inflammation, neuroendocrine activation) and systemic (e.g., stress hormones, altered circadian entrainment) responses [18], leading to a wide range of pathological alterations, such as skeletal muscle protein degradation, illness-promoting dysbiosis, and behavioral/psychological disturbances [99]. Surgical pain and post-operative nausea are known to decrease appetite and nutrient intake, thus further delaying recovery [100,101,102].
- Isolation. The unfamiliar hospital diet and sleep-wake rhythm affect the older adults in hospitals, triggering new biological routines. Limited access to visitors, role changes, and relocation have been known for decades to increase loneliness [103]. The sensorial deprivation due to limited interactions outside of the room exposes the old man to delirium along with age-associated spatial disorientation, disequilibrium, and drug-aggravated cognitive decline [90,104].

#### 3.3.2. Microgravity, Radiations, and Confinement

The spiral structure of our galaxy (the Milky Way) contains stars, planets, gas, and dust held together by gravitational forces. Our body evolved into this constant gravitational force of 1 g that has been conditioning the musculoskeletal, cardiovascular, and neuro vestibular system, thus shaping our perception of open spaces and our own body [87,105,106]. During short-term space flights, most of the body’s tissues undergo regular accommodative processes [107]. However, the further away from Earth the more the body will be exposed to various hazards that could cause mental and physical disturbances, all being problematic from both a healthy and operational viewpoint [20,24].

- Microgravity. The absence of eccentric forces exposes the body to the loss of muscle volume/strength and changes in both the composition of muscle fibers and the capillary network [108]. Along with neuromuscular deconditioning [109], the skeletal involution is the most significant alteration that arises after prolonged habitation in space, mainly deriving from the lack of both mechanical forces and sun exposure. Weightless-related conditions also affect gas exchanges and cause cardiovascular derangements, both being linked to fluid accumulation and irregular perfusion [20,110].
- Radiations. In outer space, it is not possible to grow plants in the sunlight. High-energy heavy-ion charged and solar energetic particles cause genome instabilities, carcinogenesis, tissue degeneration, and neurobehavioral disorders [111]. Unfortunately, radiation-derived oxidative stress is deleterious also for bone stability [112], further aggravating the involution of the skeleton.
- Confinement. Prolonged residence in the spaceship’s enclosed habitat exposes the astronauts to hypobaric/hypoxic situations [113] and cognitive decrements that undermine the wellbeing of the entire crew [114]. Space travelers will live in enclosed spaces with artificial illuminations, but crossing multiple time zones and exposure to different light emissions will change the circadian rhythm. The onset of mood swings and sleep disturbances can be unavoidable, being aggravated by the stressful duties [115].

Summary III. A total of 30 references ([17,18,20,24,87,90,91,92,93,95,96,97,98,99,100,101,102,103,104,105,106,107,108,109,110,111,112,113,114,115]) were used to scope the following nutritional commonalities between hospital vs. deep space hazards/illnesses. The interest in protecting the musculoskeletal system from reduced mechanical forces that expose the individual to an increased risk of fractures; the environmental consequences on the cardiovascular system; the relevance of stressful situations in causing metabolic and neurobehavioral disturbances; the potential consequences of prolonged periods of solitude.

### 3.4. Hospital-Associated vs. Space-Associated Deconditioning

#### 3.4.1. Nutritional Resilience in Orthopedic Patients

The aged orthopedic patient is a frail individual, and the hospital-associated hazards add fuel to the fire. The efficiency of the entire food system, which includes the nutritional support program, lies in the ability of the various services to communicate. If food requirements are miscalculated and nutritional sustenance is not provided, the patient’s discontent would be more likely associated with poor food intake and greater deconditioning [10]. The research laboratory should appropriately estimate energy-protein needs and the hospital food system should continuously deliver the planned food. The physiological consequences of geriatric anorexia, such as dysphagia and hyposmia [86,116], can be counteracted by food manipulation (e.g., modified texture, flavor improvement), feeding assistance, and endorsing conviviality. The excessive culinary expectations can thus be mediated. Supplements for osteosarcopenia may include diverse molecules of interest, such as β-hydroxy-β-methylbutyrate, vitamin D, calcium, and amino acids [53,117], without assuming the disconnection from other non-pharmacological interventions like daily range-of-motion exercises [118]. In the near future, most of the orthopedic care pathways will evolve, integrating on-site prescriptions with home-based monitoring after discharge, but the aforementioned hazards will persist at the patient’s home. Reduced food supply, undermined food security, and the lack of desire to feed can destabilize the nutritional status of community-dwelling lonely individuals [37]. A balanced and appetizing diet, proper meal timing, and the avoidance of foods interfering with medicines should be addressed through telemedicine interventions [90,119]. It is of paramount importance to overcome a pre-existing/hospital-derived osteosarcopenia, as it is known to be associated with poor outcome after surgery and increased risk of new fall-related traumas [120]. Undesirably, there remains uncertainty on how to defy this decay [121], and (with current knowledge) the further we move away from the peak of bone mass the more we are destined to fall.

#### 3.4.2. Nutritional Resilience in Astronauts

Many of the degenerative processes in space have many common features with the aging process on Earth [122,123], including changes in body composition, osteosarcopenia, the risk of osteoporotic fractures, nutritional frailty, hypophagia, flavor/pleasure issues, cardiovascular diseases, and neuropsychiatric derangements. As future missions will last for years, being self-sufficient is critical and so is the efficiency of the food system. Space travelers will be able to maintain good nutritional status, overcome the physiological and psychological consequences caused by a long stay in space, providing top performance every day in the most hostile environments. Considering food-interaction design, space travelers will also have to produce/process/pack their own food through novel strategies, actualizing original experiences of multisensory eating (e.g., Flavor Journey 3D Printer) [124]. The daily dietary program should meet energy and nutrient requirements, filling the deficits or reducing the intake of counterproductive substances, such as the aforementioned acidifying proteins [125]. Planning of meal timing is of great importance to regulate the sleep-wake cycle, and it can prevent the unsettling of food security or time allotted for meals [125,126]. The electrolyte imbalances and changes in splanchnic blood flow are known to be associated with delayed gastric emptying and intestinal transit [127,128], easily leading to constipation, poor assimilation of nutrients, disruption of the epithelial barrier [129], and altered gut microbiota [130]. It is, therefore, necessary to ensure a daily balance of fibers and liquids, with the possible addition of bioactive substances like caffeine, which is recommended for intense performance on Earth [131]. Nevertheless, such substances are known to interfere with critical pharmacological pathways [132] and susceptible to adverse reactions [133,134]. Defying the depletion of body reserves is also of utmost importance to prevent osteoporosis-related fractures during the transition from a low gravity field to hypergravity, which happens in the conceivable landing on unexplored planets (breaking the hip while landing on an alien planet is not recommended). It is reasonable to believe that the better the nutritional status before departure, the easier and longer the space traveler will endure the environmental conditions of space. Unfortunately, we should admit that (with current knowledge) the closer to alien worlds, the weaker the bones will be.

Summary IV. A total of 20 references ([10,33,53,90,116,117,118,119,120,121,122,123,124,125,127,128,129,130,133,134]) were used to scope the following nutritional commonalities between hospital-associated vs. space-associated deconditioning/resilience. The significance of having an efficient food system that contributes to the maintenance of the individual’s good nutritional status; the goal of reducing nutritional deficits and the intake of counterproductive substances; the importance of meal timing; the need to balance the musculoskeletal involution; the goal to avoid the recurrence of osteosarcopenia-related traumas.

## 4. Discussion

The nutritional paradigm has played a major role in human evolution since the beginning, with the *Food* factor being considered daily nourishment, sustenance during migrations, ration during explorations, fuel during skirmishes, or medicine for recovery. Any environment requires adaptation, and similar ecological hazards are known to expose humans to comparable risks and consequences. The science of Nutritional Orthopedics and Space Nutrition have individually investigated for years similar hostile environments, dealing with the aging orthopedic patient on Earth and the degenerative processes due to prolonged stay in space, respectively. Beyond the authoritative reviews on each topic that convey fundamental details [10,14,67], the purpose of this article was to show the broad spectra of the nutritional aspects that Orthopedics and Space Research have in common, focusing on the extent rather than detailed evidence. A total of 88 documents published in the last 70 years were identified, thus showing that each research field has much to share with the other. The most relevant nutrition-related matches are:

- The environmental risks, such as food insecurity, reduced movement, and stressful situations.
- The negative consequences, comprising malnutrition, osteosarcopenia, and low food intake.
- The three control phases of optimization, food security, and resilience.
- The basic strategies of a balanced diet, supplementation, and engineered food systems.

This scoping review has some limitations. First, the nature of the question may have excluded data on nutritional aspects peculiar to a single field, as unshared information was not reported. Furthermore, the research methodology may have overlooked the positive aspect of data charting among more than one reviewer, which would have ensured the accuracy of the collected data. However, the strength of this scoping review lies in the parallel mapping of two fields of research in nutrition never presented before. This method of organizing a nutritional support program into three different phases (optimization, acute phase, and resilience) could be a vital element that simplifies the comparison between any apparently diverse and complex setting. This certainly advocates not only for the need for high-quality research in each area but also for the feasibility of translating scientific evidence into different disciplines. An alignment of the shreds of evidence from Nutritional Orthopedic and Space Nutrition seems to be reasonable for mutual enrichment, with the musculoskeletal-related studies playing as communicating vessels. Back in 1959, Space Nutrition was introduced with these words: “…the scientist concerned with research in nutrition is about to be charged with the task of solving man’s nutrition problems in the milieu of space before he has more than scratched the surface in solving the problems of man in the environment of Earth” [15]. Going into space allowed us to look at our planet from another perspective. Discoveries from space researches provided broader perspectives and physiological insights to enhance different aspects of Earth-based healthcare [87], such as the abovementioned translation of hazard analysis or bed-rest studies in food industries or gerontology, respectively. However, it is also possible to exploit the nexus of learning in the opposite direction and it is, therefore, reasonable to assume that space-based tasks can be deciphered by corresponding Earth-based operative solutions. Decades of knowledge are inherited in hospitals specialized in musculoskeletal diseases and in the industries operating in the field of food supplements, modified texture foods, active packaging, and flavor enhancers. Whether we are dealing with aged orthopedic patients or space travelers, a nutrition professional specialized in the musculoskeletal system may be the common handler able to drive two trains without derailing.

## Data Availability

Not applicable.

## Supplementary Materials

The following are available online at https://www.mdpi.com/2072-6643/13/2/483/s1, Table S1. Preferred Reporting Items for Systematic reviews and Meta-Analyses extension for Scoping Reviews (PRISMA-ScR) Checklist.

## Appendix E

**Table A1:** Grouped characteristics of the sources of evidence used to map the commonalities between the Nutritional Orthopedics and Space Nutrition.

Areas of Nutritional Interest	Sources of Evidence	Topics of the Documents	Scoped Nutritional Features
***Nutritional Orthopedics***			
*Nutritional program prior to surgery*			
	[8]	Optimization of vitamin D status and cardiac function after surgery.	Vitamin D
	[10]	Perioperative nutritional program to optimize recovery.	Tailored dietary prescriptions Nutritional status
	[27]	Iron status optimization and preoperative anemia.	Iron
	[29]	Preoperative interventions to reduce recreational substances use.	Cessation/reduction of bad food habits
	[33,34]	Malnutritional status, immunocompetence, and inflammation.	Nutrition-derived immunocompetence
	[38]	Stress response and preoperative fasting vs. loading.	Food abstention
*Hospital food systems*			
	[45,46]	Attitudes of the healthcare sector, catering, and food systems.	Complex food systems Food safety
	[50]	Intake of nutrients from electronic vs. paper meal ordering system.	Demand-driven food system
	[51]	Plate waste, food costs, and meal ordering system.	Surplus disposal
*Food research laboratory in the hospital*			
	[7]	Orthopedic surgery and optimized recovery programs.	Food abstention
	[10,52,63]	Multidisciplinary nutritional programs and dietary supplements.	Advanced nutrition
	[27]	Iron status optimization and preoperative anemia.	Iron
	[53]	Geriatric osteosarcopenia and therapeutic interventions.	Calcium Proteins
	[54]	Optimization of vitamin D status and inflammation after surgery.	Vitamin D
	[55,56]	Diet, nutritional status, and interventions for osteoarthritis.	Musculoskeletal health
	[59,60,61]	Aging, hedonism, taste dysfunction, intake, and flavor enhancers.	Flavor/pleasure issues
	[62]	Facilitators of food waste, patient’s engagement, and food quality.	Meal quality Reduced food intake Food waste
*Hospital hazards*			
	[90,91]	Multifaceted aspects of geriatric syndrome.	Osteosarcopenia
	[92,93]	Mental and physical disabilities associated with hospitalization.	Low mechanical forces
	[96]	Inactivity, deterioration, and bed rest.	Fluid shifts Cardiovascular health
	[97,98]	Hospital hazards, sarcopenia, and risk of falls.	Protein/bone loss Risk of falls/fracture
	[17]	Hospital-associated deconditioning and orthostatic intolerance.	Gastrointestinal health
	[18,99]	Acute response to surgery and bacterial overgrowth in critical care.	Protein/bone loss Dysbiosis Neurobehavioral health
	[100,101,102]	Pain control, nausea, and early physical recovery.	Reduced food intake Flavor/pleasure issues
	[103]	Management of loneliness in hospitals.	Neurobehavioral health
	[90,104]	Multifaceted aspects of age-related deteriorations.	Spatial disorientation Proprioception Cognitive decrements
*Nutritional resilience in orthopedic patients*			
	[10]	Perioperative nutritional program for optimizing recovery.	Food insecurity
	[33]	Malnutritional status and immunocompetence.	Reduced food supply Food insecurity Flavor/pleasure issues
	[53,118]	Geriatric osteosarcopenia and therapeutic interventions.	Vitamin D Calcium Proteins
	[90,119]	Multifaceted geriatric syndrome and multimodal interventions.	Meal timing Flavor/pleasure issues
	[116]	Age-related anorexia and treatments.	Reduced food intake Flavor/pleasure issues
	[117]	β-hydroxy-β-methylbutyrate in older adults.	Advanced nutrition
	[120,121,123]	Hospital-associated deconditioning, interventions, and outcomes.	Osteosarcopenia Risk of falls/fracture
*Space Nutrition*			
*Nutritional conditioning prior to launch*			
	[39]	Food to sustain life during early ventures in space.	Nutritional status
	[40]	Immunocompetence, in-flight supplementation, and workout.	Nutrition-derived immunocompetence
	[19]	Food, beverage, and menus in space.	Tailored dietary prescriptions
	[25]	Aeromedical factors and pilot’s health and performance.	Cessation/reduction of bad food habits
	[20]	Astronaut’s health and performance.	Food abstention
	[42]	Food and nutrition sciences for exploration missions in space.	Advanced nutrition Food safety
*Space food systems*			
	[21,64,67]	History of space food, system development, technical goals.	Complex food systems
	[66,67]	History of space plant growth systems, development trends.	Demand-driven food system Reduced food supply Surplus disposal
	[22,68,73]	Challenges for sustainable systems in space, insects as foods.	Advanced nutrition
*Food research laboratory in space*			
	[20]	Astronaut’s health and performance.	Flavor/pleasure issues
	[23,64,67]	History of space food, system development, food science goals.	Meal quality Food waste
	[76,77]	Food growth and nutrient performance in space.	Advanced nutrition
	[79]	Analysis of nutrient contents in processed space foods.	Food insecurity
	[82]	Interventions for bone loss during long-term spaceflight.	Vitamin D Calcium Musculoskeletal health
	[83,85]	Musculoskeletal involution and other space-related issues.	Proteins Musculoskeletal health
	[84]	Microgravity and radiation, musculoskeletal health, iron status.	Iron
*Space hazards*			
	[20,110]	Astronaut’s health, performance, and cardiac function.	Fluid shifts Cardiovascular health
	[95]	The role of the constant and pervasive force of gravity.	Low mechanical forces
	[87,105,106]	Translation of space research, bodily affections of microgravity.	Proprioception Spatial disorientation
	[24,107,108,109]	Musculoskeletal remodeling, space hazards, countermeasures.	Musculoskeletal health
	[111]	Biological affections from space radiation exposure.	Neurobehavioral health
	[112]	Mechanisms of the oxidative stress-induced involution in space.	Protein/bone loss
	[113]	Physiological adjustments to hostile environments.	Reduced food intake
	[114]	Dietary intakes, requirements, and psychosocial aspects in space.	Cognitive decrements
	[115]	Immune dysfunction, countermeasures for exploration missions.	Neurobehavioral health
*Nutritional resilience in space travellers*			
	[122]	Changes induced by models of microgravity and osteosarcopenia.	Osteosarcopenia
	[124]	Comprehensive human experience of eating in space.	Flavor/pleasure issues
	[125]	Malnutrition after long-term space flight.	Nutritional statusMeal timingFood insecurity
	[127,128,129,130]	Gastrointestinal affections of microgravity and research goals.	Gastrointestinal health Dysbiosis
	[133,134]	Performance risks associated with supplements in aviation.	Food safety

## Appendix F

**Table A2:** Characteristics of individual sources of evidence included in the scoping review.

Areas of Nutritional Interest	Author, Year	Editorial Source, Document Type	Population or Setting/Findings
***Nutritional Orthopedics***			
*Nutritional program prior to surgery*			
	[8] Briguglio, 2018	J Geriatr Cardiol, Human study	Orthopedic patients/Vitamin D supplementation may be associated with an amelioration of cardiac function and hemodynamic.
	[10] Briguglio, 2019	Nutr Clin Metab, Review	Orthopedic patients/A nutritional program accounting for preoperative, postoperative, and support after discharge should be integrated.
	[27] Briguglio, 2020	Nutrients, Human study	Orthopedic patients/Oral iron can optimize the preoperative iron status, with gastrointestinal diseases and medications affecting efficacy.
	[29] Sarin, 2017	J Surg Oncol, Review	Hospital environment/Oral clear fluid intake or carbohydrate-based beverages should be liberalized prior to elective surgery.
	[33] Budworth, 2019	Front Psychol, Meta-analysis	Hospital environment/Alcohol or other recreational substance use should be reduced by targeted preoperative interventions.
	[34] Durrand, 2019	Clin Med (Lond), Review	Hospital environment/Prehabilitation principles interest psychology, exercise, nutrition, alcohol, smoking, and patient’s education.
	[38] Mau, 2018	Exp Gerontol, Review	Older adults/Adipose tissue inflammation is associated with impaired metabolic health, energy storage, and lipid metabolism.
*Hospital food systems*			
	[45] Hofer, 2013	Int J Facil Manag, Public health study	Hospitalized patients/Cost transparency, benchmarking activities, and cost of meals per patient in hospitals need to be examined.
	[46] Edwards, 2006	J Hum Nutr Diet, Human study	Hospitalized patients/Novel food systems may improve food intakes and satisfaction along with reduced wastage at the ward level.
	[50] Maunder, 2015	Clin Nutr ESPEN, Human study	Hospitalized patients/Dietary intake and satisfaction may be enhanced by an electronic meal ordering system compared to a paper menu.
	[51] McCray, 2018	Clin Nutr ESPEN, Human study	Hospitalized patients/Food waste, patient’s engagement, and costs can be controlled by an electronic bedside meal ordering system.
*Food research laboratory in hospital*			
	[7] Kaye, 2019	J Anaesthesiol Clin Pharmacol, Review	Hospital environment/Enhanced recovery pathways consist of perioperative interventions to improve outcomes and reduce stay.
	[10] Briguglio, 2019	Nutr Clin Metab, Review	Orthopedic patients/A nutritional program accounting for preoperative, postoperative, and support after discharge should be integrated.
	[27] Briguglio, 2020	Nutrients, Human study	Orthopedic patients/Oral iron can optimize the preoperative iron status, with gastrointestinal diseases and medications affecting efficacy.
	[52] Rask, 2020	Mil Med, Public health study	Orthopedic patients/Multivitamin supplementation may enhance the recovery, but dosages and duration should be investigated.
	[53] Fatima, 2019	Adv Musculoskelet Dis, Review	Older adults/Nonpharmacological strategies, such as nutrition and exercise, may be considered for treating osteosarcopenia.
	[54] Briguglio, 2020	Nutr Clin Metab, Human study	Orthopedic patients/The correction of hypovitaminosis D is associated with a reduction of inflammation and endothelial dysfunction.
	[55] Purcell, 2016	Can J Diet Pract Res, Human study	Orthopedic patients/Before and after surgery, targeted dietary and exercise interventions may be useful in patients with osteoarthritis.
	[56] Morales-Ivorra, 2018	Nutrients, Review	Orthopedic patients/There may be an association between the onset of osteoarthritis and the adherence to a Mediterranean dietary pattern.
	[59] Joussain, 2013	PLoS One, Human study	Older adults/Normal aging is associated with reduced odor perception, possibly related to changes in pleasant odor processing.
	[60] Iannilli, 2017	J Neurosci Res, Human study	Older adults/Age-related reduction of taste may be due to both central and peripheral degradation of neural signatures.
	[61] Mathey, 2001	J Geron A Biol Sci Med Sci, Human study	Older adults/Dietary intake and body weight in institutionalized older adults may be improved by adding flavor boosters to foods.
	[62] Schiavone, 2019	Int J Envir Res Pub Health, Human study	Hospitalized patients/Quality improvement of meal reservation, accounting specific preferences, is promising for plate waste reduction.
	[63] Bell, 2014	Clin Nutr, Human study	Orthopedic patients/Multimodal nutritional support plus foodservice enhancement increase food intakes and improve outcomes.
***Hospital hazards***			
	[90] Briguglio, 2020	Front Psychol, Opinion	Older adults/Home-confinement generates a more fragile class of older adults, with long-term psychological consequences.
	[91] Belchior, 2020	Eur Geriatr Med, Review	Older adults/Early interventions, like resistance exercise and adequate protein, vitamin D, calcium, may delay osteosarcopenia onset.
	[92] Covinsky, 2011	JAMA, Review	Hospital environment/Hospitalization-associated disability may be prevented towards functional status studies in acute geriatric units.
	[93] Friedman 2008	Gerontologist, Human study	Hospitalized patients/Hospitalization is associated with high risks for falls and functional decline. Targeted programs should be developed.
	[96] Corcoran, 1991	West J Med, Review	Hospital environment/Inactivity damages each of the body’s organ systems similarly to aging. Preventive strategies should be planned.
	[97] Creditor, 1993	Ann Intern Med, Review	Hospital environment/De-emphasizing bed rest and actively facilitating ambulation and socialization can avoid the dependency cascade.
	[98] Landi, 2012	Clin Nutr, Human study	Older adults/Sarcopenia is highly prevalent, leading to greater risks for fall, regardless of age, gender, or other confounding factors.
	[17] Han, 2018	Elsevier, Book chapter	Hospitalized patients/Bed rest should be avoided. Early mobilization should be promoted to counteract multiple system dysfunction.
	[18] Hashmi, 2018	Cambridge, Book chapter	Hospitalized patients/The adaptation to stress impairs with aging. Surgical trauma in older adults is associated with impaired recovery.
	[99] McDonald, 2016	mSphere, Human study	Hospitalized patients/Health-promoting microbiome signatures may guide to therapeutic interventions in critical illnesses.
	[100] Eriksson, 2017	J Adv Nurs, Human study	Orthopedic patients/It is critical to monitor a patient’s pain. It is associated with physical recovery after surgery and with general outcomes.
	[101] Eriksson, 2019	J Adv Nurs, Human study	Orthopedic patients/It is important to monitor postoperative nausea. The simple measure of intensity is associated with physical recovery.
	[102] Falzone, 2013	Drugs Aging, Review	Older adults/For most analgesic strategies, “start low and go slow” should be adopted in view of pharmacokinetic-dynamic changes.
	[103] Rodgers, 1989	J Gerontol Nurs, Opinion	Hospitalized patients/Nurses, through ensuring access to visitors and facilitating involvement in activities, may decrease loneliness.
	[104] Dunsky, 2019	Front Aging Neurosci, Review	Older adults/Dual-task function-oriented challenges along with balance control can stimulate the sensory-neuromuscular controls.
*Nutritional resilience in orthopedic patients*			
	[10] Briguglio, 2019	Nutr Clin Metab, Review	Orthopedic patients/A nutritional program accounting for preoperative, postoperative, and support after discharge should be integrated.
	[33] Budworth, 2019	Front Psychol, Meta-analysis	Hospital environment/Alcohol or other recreational substance use should be reduced by targeted preoperative interventions.
	[53] Fatima, 2019	Adv Musculoskelet Dis, Review	Older adults/Nonpharmacological strategies, such as nutrition and exercise, may be considered for treating osteosarcopenia.
	[90] Briguglio, 2020	Front Psychol, Opinion	Older adults/Home-confinement generates a more fragile class of older adults, with long-term psychological consequences.
	[116] Landi, 2016	Nutrients, Review	Older adults/Age-related anorexia negatively influences appetite and food intake. Oral supplements or modified diets should be studied.
	[117] Oktaviana, 2019	J Nutr Health Aging, Review	Old patients/The impairment of muscle mass and strength in sarcopenia or frailty may be preserved by β-hydroxy-β-methylbutyrate.
	[118] Kirk, 2020	J Cachexia Sarcopenia Muscle, Editorial	Older adults/Osteosarcopenia is an emerging geriatric syndrome and the preventive maximization of musculoskeletal health is mandatory.
	[119] Briguglio, 2020	Neuropsychiatr Dis Treat, Opinion	Older adults/Healthy eating, exercise, and sleep are critical components to prevent and manage neuropsychiatric disorders.
	[120] Bae, 2020	Ger Orthop Surg Rehab, Human study	Orthopedic patients/Osteoporosis is correlated to sarcopenia. Osteosarcopenia is associated with poor outcomes and high fracture rates.
	[121] Smith, 2020	Arch Gerontol Geriatr, Meta-analysis	Hospitalized patients/There is the need for more studies on enhanced programs targeted at hospital-associated deconditioning.
	[123] Paintin, 2019	Br J Hosp Med (Lond), Review	Older adults/Osteosarcopenia is associated with individual falls, fractures, mortality, and socioeconomic burden for the society.
***Space Nutrition***			
*Nutritional conditioning prior to launch*			
	[39] Unknown, 1960	Nutr Rev, Review	Space environment/Advanced inactivation methods, packaging, and lightweight food systems are critical points to sustain health in space.
	[40] Baba, 2020	Nutrients, Human study	Healthy individuals/The space diet lacks omega-3 fatty acids, β-alanine, and carnosine. Supplementation should be considered.
	[19] Casaburri, 1999	NASA, Guide	Space environment/In-depth knowledge about food science and technology is required to preserve and package food for space.
	[25] FAA, 2016	FAA, Book chapter	Pilots/Flying requires mental and physical standards to resist hypoxia, spatial disorientation, motion sickness, and other major issues.
	[20] Lane, 2010	NASA, Book chapter	Space environment/Research and health care are critical factors during shuttle flights and missions beyond low-Earth orbit.
	[42] Douglas, 2020	J Nutr, Opinion	Space environment/Space food should meet safety, stability, palatability, variety, reliability, resource minimization, usability, nutrition.
*Space food systems*			
	[64] Perchonok, 2002	Nutrition, Review	Space environment/Extended space missions need nutritious and easily consumable food along with advanced crops for food security.
	[66] Zabel, 2016	Life Sci Space Res (Amst), Review	Space food systems/Novel agriculture technologies for bio-regenerative life support systems can enable long-term space missions.
	[67] Oluwafemi, 2018	Adv Astronaut Sci Technol, Review	Space environment/Knowledge on plant growth, methods to simulate soils, and greenhouse facilities should be addressed for space.
	[21] Massa, 2018	NASA, Technical report	Vegetable-production system/Advanced food systems can grow fresh vegetables, helping to mitigate the risk of inadequate supplies.
	[68] Carillo, 2020	Agron J, Review	Space food systems/Bio-regenerative life support systems should fulfill physical constraints and energy requirements for space farming.
	[22] Dufour, 1981	NASA, Technical report	Insects/Insects are a potential food source in space, providing a stream of proteins for sustaining astronauts’ health.
	[73] Tong, 2011	Bull Entomol Res, Animal study	Insects/To feed silkworms with inedible biomass is a promising approach for providing high-quality proteins for astronauts.
*Food research laboratory in space*			
	[20] Lane, 2010	NASA, Book chapter	Space environment/Research and health care are critical factors during shuttle flights and missions beyond low-Earth orbit.
	[64] Perchonok, 2002	Nutrition, Review	Space environment/Extended space missions need nutritious and easily consumable food along with advanced crops for food security.
	[67] Oluwafemi, 2018	Adv Astronaut Sci Technol, Review	Space environment/Knowledge on plant growth, methods to simulate soils, and greenhouse facilities should be addressed for space.
	[23] Brief, 2015	NASA, Online report	Space food systems/Surplus disposal, reduced mass/volume of the food system, and extended shelf life are vital research goals for space.
	[76] Khodadad, 2020	Front Plant Sci, Food study	Lettuce/Leafy vegetables from the Veggie plant growth units can provide fresh and safe food to integrate the astronauts’ diet.
	[77] Yuan, 2017	Aquat Bot, Food study	Duckweed/Simulated microgravity does not significantly affect duckweed growth, ultra-structure, and starch content.
	[79] Cooper, 2017	NPJ Microgravity, Food study	Space food/Space food stored over 3 years encounter nutritional impoverishment. Advanced methods should stabilize nutrient content.
	[82] Smith, 2012	J Bone Miner Res, Human study	Astronauts/Improving nutrition and resistance exercise in space can mitigate the musculoskeletal involution over 6-month missions.
	[83] Hackney, 2014	Life (Basel), Review	Astronauts/To protect musculoskeletal health and to increased protein/amino acid intakes with exercise may be promising.
	[84] Yang, 2018	Int J Mol Sci, Review	Astronauts/Iron overload and oxidative damage are deleterious effects of space. Iron chelators and antioxidants should be investigated.
	[85] Stein, 2001	Nutr Res Rev, Review	Astronauts/Space habitation is associated with musculoskeletal remodeling, protein and bone loss, and reduced dietary intakes.
***Space hazards***			
	[20] Lane, 2010	NASA, Book chapter	Space environment/Research and health care are critical factors during shuttle flights and missions beyond low-Earth orbit.
	[87] Shelhamer, 2020	NPJ Microgravity, Perspective	Space environment/Findings from space research, like the research results from bone physiology, have enhanced healthcare on Earth.
	[95] Page, 1977	N Engl J Med, Review	Space environment/Physiologic and morphologic changes in animals raised in weightlessness should be a prospect for future research.
	[105] Ferrè, 2019	Sci Rep, Human study	Healthy individuals/The perception of weight depends on immediate sensory signals after an experimentally altered gravitation.
	[106] Bizzarri, 2015	BioMed Research International, Editorial	Space environment/More appropriate experimental models are needed to study the gravity-related changes of cellular dynamics.
	[107] Stein, 2013	Eur J Appl Physiol, Perspective	Space environment/Energy, muscle, bone issues may have been exaggerated. More studies on musculoskeletal involution are needed.
	[110] Palacios, 2019	Front Physiol, Human study	Healthy individuals/Microgravity is associated with an increased risk of repolarization instabilities and arrhythmias.
	[24] Abadie, 2020	NASA, Online document	Astronauts/Space hazards are gravity, isolation, confinement, radiation, and distance. Their effects should be minimized.
	[108] Fitts, 2010	J Physiol, Human study	Astronauts/Prolonged stay in space causes loss of muscle fiber mass, force, and power. Exercise countermeasures should be improved.
	[109] English, 2019	Compr Physiol, Review	Astronauts/It is vital to study physiological thresholds below which task performance is reduced along with exercise countermeasures.
	[111] McDonald, 2020	Cancers (Basel), Bioinformatics study	Animal models/Novel hypotheses on the human risks from space radiations can be generated from the GeneLab’s rich database.
	[112] Tian, 2017	Int J Mol Sci, Review	Space environment/Radiations and microgravity in space can elicit oxidative stress in humans, causing a deleterious skeletal involution.
	[113] Pasiakos, 2020	Annu Rev Nutr, Review	Hostile environment/Extreme temperatures and high-altitude alter expenditures and intakes, not fully met by dietary recommendations.
	[114] Lane, 1994	Am J Clin Nutr, Review	Astronauts/Dietary intakes may be lower than predicted, impacting the endocrine, immune, musculoskeletal, and psychosocial systems.
	[115] Crucian, 2018	Front Immunol, Review	Space environment/It is important to study immune countermeasures in space, which comprise supplements, foods, exercise, or drugs.
*Nutritional resilience in space travelers*			
	[122] Tomilovskaya, 2019	Front Physiol, Review	Space environment/Dry immersion accurately simulates factors of space flight, reproducing the full spectrum of bodily changes.
	[124] Obrist, 2019	Front Comput Sci, Human/Food study	Healthy individuals/Food has a multi-dimensional and -sensorial role, and the eating experiences in space should account for all these areas.
	[125] Smith, 2005	J Nutr, Human study	Astronauts/After an extended stay in space, nutritional changes occur, comprising bone loss, poor vitamin D status, and oxidative damage.
	[127] Amidon, 1991	J Clin Pharmacol, Review	Space environment/The absence of gravity may negatively affect gastric emptying, intestinal transit, and drug absorption.
	[128] Millet, 2001	Clin Physiol, Human study	Healthy individuals/Controlled physical inactivity during head-down bed rest caused altered sympathetic activity and protein metabolism.
	[129] Alvarez, 2019	Sci Rep, In vitro study	Astronauts/Gastrointestinal homeostasis in space encounters a protracted susceptibility to epithelial barrier disruption.
	[130] Bergouignan, 2016	NPJ Microgravity, Review	Space environment/Space research interests include energy balance, eating, metabolic stress, deficiencies, dysbiosis, and fluid balance.
	[133] Barker, 2011	Aviat Space Environ Med, Case report	Pilots/G tolerance can be altered by dietary supplements in turn potentially causing a catastrophic G-induced loss of consciousness.
	[134] Sather, 2013	The Sport Journal, Review	Pilots/Military aviators may use dietary supplements to enhance performance, but safety and effectiveness should be regulated.
